# Sleep in 21-Day Dry Immersion. Are Cardiovascular Adjustments Rapid Eye Movement Sleep-Dependent?

**DOI:** 10.3389/fphys.2021.749773

**Published:** 2021-10-26

**Authors:** Evgeny Yu. Bersenev, Yulia V. Ukraintseva, Gennadiy V. Kovrov, Yusef D. Yakhya, Galina Yu. Vassilieva, Elena S. Tomilovskaya, Ilya V. Rukavishnikov, Sergey I. Posokhov, Artemiy V. Orlov, Nikolay Yu. Osetsky, Oleg I. Orlov

**Affiliations:** ^1^State Scientific Center of the Russian Federation Institute of Biomedical Problems of the Russian Academy of Sciences (SSC RF IBMP RAS), Moscow, Russia; ^2^Institute of Higher Nervous Activity and Neurophysiology of the Russian Academy of Sciences (IHNA&NPh RAS), Moscow, Russia; ^3^Biomedical Science & Technology Park of I.M. Sechenov First Moscow State Medical University of the Ministry of Health of the Russian Federation (Sechenov University), Moscow, Russia

**Keywords:** dry immersion, sleep, arterial blood pressure, cardiovascular system, rapid eye movement sleep, cortisol

## Abstract

**Introduction:** A decrease in sleep quality and duration during space missions has repeatedly been reported. However, the exact causes that underlie this effect remain unclear. In space, sleep might be impacted by weightlessness and its influence on cardiovascular function. In this study, we aimed at exploring the changes of night sleep architecture during prolonged, 21-day Dry Immersion (DI) as one of the ground-based models for microgravity studies and comparing them with adaptive changes in the cardiovascular system.

**Methods:** Ten healthy young men were exposed to DI for 21 days. The day before (baseline, B-1), on the 3rd (DI3), 10th (DI10), and 19th (DI19) day of DI, as well as in the recovery period, 1 day after the end of DI (R + 1), they were subjected to overnight polysomnography (PSG) and ambulatory blood pressure monitoring.

**Results:** On DI3, when the most severe back pain occurred due to the effects of DI on the spine and back muscles, the PSG data showed dramatically disorganized sleep architecture. Sleep latency, the number of awakenings, and the duration of wake after sleep onset (WASO) were significantly increased compared with the B-1. Furthermore, the sleep efficiency, duration of rapid eye movement sleep (REM), and duration of non-rapid eye movement stage 2 decreased. On DI10, subjective pain ratings declined to 0 and sleep architecture returned to the baseline values. On DI19, the REM duration increased and continued to rise on R + 1. An increase in REM was accompanied by rising in a nighttime heart rate (HR), which also shows the most significant changes after the end of DI. On DI19 and R + 1, the REM duration showed opposite correlations with the BP parameters: on DI19 it was negatively associated with the systolic BP (SBP), and on R + 1 it was positively correlated with the diastolic BP (DBP).

**Conclusion:** An increase in REM at the end of DI and in recovery might be associated with regulatory changes in the cardiovascular system, in particular, with the reorganization of the peripheral and central blood flow in response to environmental changes.

## Introduction

A decrease in sleep quality and quantity during space missions has repeatedly been reported (Polyakov et al., 1994; Gundel et al., 1997; Barger et al., 2014). Already the first studies using polysomnography (PSG) in space showed alterations in sleep architecture (Frost et al., 1975, 1976; Gundel et al., 1997). For example, PSG recordings performed aboard the Russian space station Mir showed a decreased total sleep time. In addition, the sleep architecture was significantly altered: the latency of rapid eye movement sleep (REM) was shorter, and slow-wave sleep (SWS) was redistributed from the first to the second sleep cycle (Gundel et al., 1997). However, the exact causes that underlie this effect remain unclear.

Exposure to weightlessness is accompanied by profound changes in most physiological systems, including disturbances in the sensorimotor, skeletal, and muscular systems, and, first of all, changes in the cardiovascular system (LeBlanc et al., 2000; Eckberg, 2003). In space, weightlessness immediately induces an upward fluid shift (Thornton and Hoffler, 1977). This fluid shift initiates subsequent changes in the cardiovascular system, including changes in the arterial and venous hemodynamics and vascular tone (Gazenko, 1984; Norsk et al., 2015). The redistribution of fluids also includes a decrease in the plasma volume (Watenpaugh and Hargens, 1996). Thus, cardiovascular deconditioning caused by weightlessness might be one of the predisposing factors for sleep disturbance.

Under the conditions of gravity, one of the most comparable models of weightlessness in terms of physiological effects is Dry Immersion (DI) (Tomilovskaya et al., 2019). The immersion is called “dry” because a waterproof film separates the subject from water. Since its development, DI has been the basic model in Russia for studying microgravity effects. The DI reproduces three main effects of weightlessness: physical inactivity, support withdrawal, and elimination of the vertical vascular gradient. Like weightlessness, the DI causes central hypervolemia with a subsequent decrease in the plasma volume and associated increased diuresis (Leach Huntoon et al., 1998; Noskov et al., 2011). The DI also reproduces vertebral deconditioning repeatedly reported after spaceflight (Treffel et al., 2020).

The first study on the effects of DI on sleep quality was based on an analysis of subjective reports of participants (Slonov et al., 2017). Subjective ratings showed disturbed sleep in the first days of DI when volunteers experienced back pain due to spinal lengthening. However, after 3–4 days of DI, they complained less about pain and discomfort and noted better sleep quality. Unfortunately, PSG studies of sleep in DI have not been carried out yet, and the effects of DI on sleep architecture remain unstudied so far.

Therefore, based on the literature data, we hypothesized that after the first days of DI sleep gradually becomes consolidated and, using PSG in prolonged, 21-day immersion, specific effects of hypogravity on sleep architecture might be investigated. Considering the stress-protecting role of sleep (Vgontzas et al., 2000), we decided to determine whether any feature of sleep architecture could be associated with cardiovascular adjustments during acclimation to the DI and recovery.

## Materials and Methods

### Participants

After providing written informed consent 10 healthy male volunteers aged from 23 to 34 (mean age 29.3 ± 3.8 years; height 176.4 ± 3.8 cm; weight 71 ± 10.6 kg; body mass index 22.7 ± 2.7) participated in the study. All participants had no history of cardiovascular disease and took no medication. The study was conducted in accordance with the declaration of Helsinki. The protocol was approved by the ethics commissions of the Institute of Biomedical Problems (Moscow) (protocol no. 483 dated August 3, 2018).

Study eligibility was determined via written questionnaires about sleep quality, habitual sleep time, daytime sleepiness, physical and mental health, medication use, and health behaviors (e.g., smoking, alcohol consumption, and work and study schedules). The exclusion criteria included a history of head injury, chronic or acute illness, current medication of any kind, alcohol or drug abuse, smoking, shift work, excessive daytime somnolence, sleep complaints, and the presence of any oral inflammatory processes with or without evident bleeding. Participants were required to maintain a strict regular sleep-wakefulness schedule for 7 days prior to Dry Immersion start with bedtimes between 23:00 and 24:00 h, wake-up times between 07:00 and 08:00 h, as well as refrain from taking naps during the day.

### Study Design

The DI was performed with the use of specialized waterproof and highly elastic fabric. A participant of the study wearing a T-shirt and trunks was put on waterproof fabric and immersed into a deep bath up to the neck in a supine position. The area of the fabric surface considerably exceeded those of the water surface. The folds of the waterproof fabric allowed the person body to be enveloped from all sides freely. During the DI exposure, the participants were placed individually in a supine position in a bath with dimensions of 200 × 100 × 100 cm. The bath was filled with water at the temperature maintained constant at 33 ± 0.5°C. The daily routine was specified following the schedule of studies, including night sleep, 3 meals, a medical supervision program, and experimental studies without daytime naps (a schedule of the day preceding polysomnography is presented in Supplementary Material). The research participants were taken out of the DI for 15–20 min each day for sanitary and hygienic procedures by the use of a specialized lift rising from the bottom of the bath.

Objective sleep architecture evaluation was performed by overnight polysomnography (PSG) at the baseline 1 day before the start of DI (B-1), at the 3rd (DI3), 10th (DI10), and 19th (DI19) days of DI, as well as after the first day of recovery (R + 1). Nighttime blood pressure (BP) monitoring and electrocardiogram (ECG) recordings were conducted simultaneously with the PSG. Measurements started 30 min before the time for lights out and ended 1 h after lights were on.

On the B-1 and R + 1, taking into account individual preferences, the time for lights out ranged from 23.00 to 24.00 and the time for lights on was from 07.00 to 08.00. During all 21 days of DI, the time for lights out ranged from 22.30 to 23.30 and that for lights on from 07.30 to 08.30. Thus, the sleep opportunity on the B-1 and R + 1 was 8 h and during DI it was 9 h. Sleep opportunity was longer during DI, considering the participants needed longer time in bed to recover due to discomfort associated with the DI procedure (e. g., increased urination, including at night).

### Signal and Data Acquisition and Analysis

#### Polysomnographic Data Acquisition and Scoring

PSG recordings were performed using a Neuron-Spectrum digital EEG amplifier (Neurosoft Company, Ivanovo, Russia) with a sampling rate of 250 Hz. The PSG recordings included an EEG (F3, F4, C3, C4, O1, and O2 placed in accordance with the International 10–20 System), electrooculogram, electromyogram, airflow, thoracic, and abdominal movements. Polysomnograms were scored offline by two scorers who were blinded to the experimental conditions. Visual scoring of every 30-s epoch of PSG recording as wakefulness, non-rapid eye movement sleep (NREM) stage N1, N2, N3 (slow-wave sleep/SWS), or REM was performed according to the standard AASM criteria (Iber et al., 2007). The interscorer reliability was > 93.5%. The PSG variables analyzed included sleep onset latency, the latency of SWS, the latency of REM, total sleep time (TST), number of awakenings, wakefulness after sleep onset (WASO), sleep period time (SPT), and sleep efficiency. The SPT was measured as the period beginning from the moment when the participant fell asleep and ending at the last wake-up, including the duration of awakenings. The sleep efficiency was calculated as a percent value of TST referred to as time in bed (TIB). We also used N1, N2, SWS, and REM durations as a percentage of SPT.

#### Cardiovascular Monitoring

For the BP measuring we used a BpLab ambulatory BP monitor (Nizhny Novgorod, Russia). A cuff was applied to the volunteer left shoulder connected by a flexible hose to the device attached to the waist belt. Measuring was carried out every 60 min. An analysis of the BP data was performed using the BpLab software.

The ECG recording (Holter ECG monitoring) was carried out in three leads using a HolterLive portable wireless monitor (Softest ATE Ltd., Kursk). Electrodes were fixed in the projection of the apex of the heart. An analysis of the ECG data was performed using an original software.

For statistical analysis, we used BP and HR values averaged over the whole night.

#### Glucocorticoid Measurements

Samples of mixed saliva for analysis of cortisol levels were collected in Salivette^®^ tubes (Sarstedt, Germany) on the days of polysomnography two times a day: in the evening 30 min before the time for lights out and in the morning after awakening. The samples were immediately centrifuged and stored at –80°C. After thawing, aliquots were analyzed by enzyme-linked immunosorbent assay (ELISA) using commercial test systems [Diagnostics Biochem Canada Inc. (DBC)].

#### The Subjective Pain and Discomfort Assessment Instruments

The subjects were asked about the localization of pain and its intensity using the Digital Rating Scale method (NRS) (Karoly and Jensen, 1987). The digital rating scale consists of 11 points: from 0, no pain; to 10, pain that cannot be tolerated. Measurements were performed within 7 days before the start of the experiment, daily during DI exposure (three times a day with subsequent averaging), and 2, 24, 48, and 72 h after the end of DI. As the baseline values, we used averaged values obtained over 7 days before the DI start. We compared them with the daily data during DI and daily data in the recovery.

#### Statistical Analysis

The data analyses were performed using the Statistica 10 software (Stat Soft. Inc., Tulsa, OK, United States). The data were evaluated for a normal distribution using the Kolmogorov–Smirnov test. A repeated-measures analysis of variance (rANOVA) was conducted to examine differences in the PSG, heart rate, blood pressure (BP), and cortisol concentrations. For *post hoc* analyses, the Fisher LSD test was performed. The Wilcoxon test was used to compare the data that were not normally distributed. Pearson’s correlation analysis was conducted to assess the correlations between the BP, heart rate, and REM duration. The statistical significance was set at *p* < 0.05 (Lang and Secic, 2016).

## Results

**FIGURE 1:**
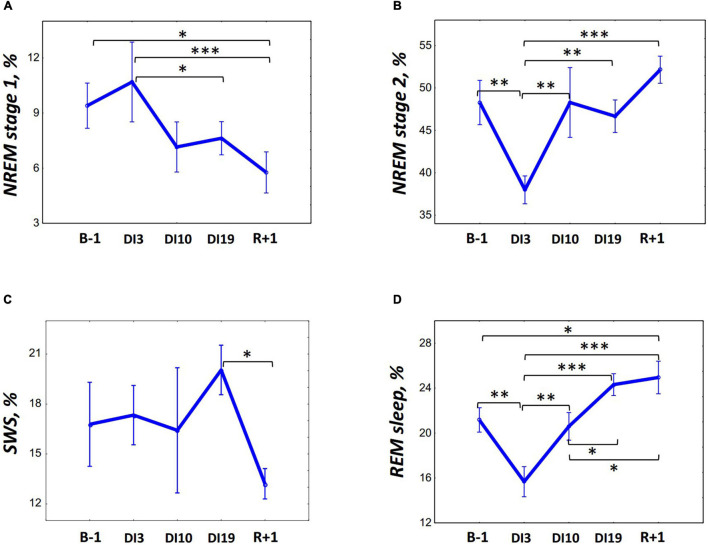
Changes in the sleep architecture characteristics during 21-day Dry Immersion and recovery from it. **(A)** NREM stage 1, stage 1 of non-rapid eye movement sleep. **(B)** NREM stage 2, stage 2 of non-rapid eye movement sleep. **(C)** SWS, slow-wave sleep. **(D)** REM, rapid eye movement sleep. B-1, the day before dry immersion, DI3, DI10, DI19, on the 3rd, 10th, and 19th days of dry immersion, respectively, R + 1, 1 day after the end of dry immersion. The data presented are a percentage of the sleep period time, means ± SEM. Intersession differences were examined using the *post hoc* Fisher LSD test. ^∗^, ^∗∗^, ^∗∗∗^ represent significance *p* < 0.05, *p* < 0.01, *p* < 0.001, respectively. Photo of the volunteer during the ambulatory blood pressure monitoring and polysomnography recording in 21-day Dry Immersion. Photo by O.G. Voloshin (IBMP).

On DI3, the PSG data showed dramatically disorganized sleep (Table 1). Unfortunately, when analyzing the data, we found that on DI3, some study participants had short sleep episodes after the wake time, which occurred within 1 h after lights on. It was a private time for our participants, and they were not strictly controlled at this period. Thus, the TIB on DI3 exceeded the TIB in other DI sessions. Despite this, we decided not to exclude these data from the analysis since they are also an essential feature of sleep in DI. When compared with the baseline, on DI3 there was a significant increase in the number of awakenings [*F*_(__4, 36)_ = 4.70, *p* = 0.004; *post hoc* relative to the B-1: *p* = 0.002], WASO [*F*_(__4, 36)_ = 6.52, *p* < 0.001; *post hoc* relative to the B-1: *p* < 0.001], sleep latency [*F*_(4, 36)_ = 3.85, *p* = 0.011; *post hoc* relative to the B-1: *p* = 0.004], and a decrease in sleep efficiency [*F*_(__4, 36)_ = 2.14, *p* = 0.096; *post hoc* relative to the B-1: *p* < 0,010]. Also we found a statistically significant increase in SWS on DI3 compared with the B-1 (*p* < 0.033) and R + 1 [*p* < 0.002, *F*_(__4, 36)_ = 3.53, *p* = 0.016]. However, since the time in bed on DI3 was the longest, an analysis of percentages seems to be more appropriate. An analysis of the sleep stage durations as a percentage of SPT showed that on DI3 the percentage of REM [*F*_(4, 36)_ = 8.71, *p* < 0.001; *post hoc* relative to the B-1: *p* = 0.004] and N2 [*F*_(4, 36)_ = 6.10, *p* = 0.001; *post hoc* relative to the B-1: *p* = 0.002] were significantly lower than those in the baseline (Figure 1). On DI10, sleep, on the whole, returned to the baseline values: the number of awakenings and WASO were still slightly increased but no longer differed significantly from the B-1. On DI19, most of the PSG characteristics were comparable to the baseline values. Except for the REM percentage, which increased and became marginally significantly higher than that on B-1 (*p* = 0.084), and significantly higher than that on DI10 (*p* = 0.043). On R + 1, the PSG data showed a high sleep efficiency: WASO, the number of awakenings, and sleep latency did not differ from the baseline, and the percentage of N1 was even significantly lower than that on B-1 (*p* = 0.022). At the same time, the REM percentage was increased (*post hoc* relative to the B-1: *p* = 0.039; relative to the DI3: *p* > 0.001; and relative to the DI10: *p* = 0.019), and the SWS percentage reduced (*post hoc* relative to the DI19: *p* = 0.027).

**TABLE 1:** Mean values of polysomnographic data in all sessions.

	**B-1**		**DI3**		**DI10**		**DI19**		**R + 1**	
	**Mean**	**SE**	**Mean**	**SE**	**Mean**	**SE**	**Mean**	**SE**	**Mean**	**SE**

Total bed time (min)	480.00	0	585.60	12.81	540.00	0	540.00	0	480.00	0
Total sleep time (min)	416.53	8.98	456.67	17.39	443.50	24.43	441.67	27.53	439.18	11.54
Sleep period time (min)	433.72	10.61^#^	573.83	39.27*^$&@^	484.50	28.62^#^	454.17	27.44^#^	457.79	13.91^#^
WASO (min)	17.19	4.99^#^	117.17	32.55*^$&@^	41.00	19.38^#^	12.50	4.60^#^	18.61	5.35^#^
Sleep efficiency (%)	0.86	0.02	0.78	0.02^@^	0.82	0.05	0.82	0.05	0.92	0.02^#^
Sleep onset latency (min)	5.31	1.86^#^	29.08	11.69*^$&@^	5.80	3.07^#^	6.00	1.32^#^	3.89	1.17^#^
Latency of SWS (min)	30.94	6.77	48.50	10.61	42.00	17.37	42.28	20.85	22.39	3.09
Latency of REM (min)	98.06	15.98	126.67	46.41	101.55	22.71	83.83	17.36	74.11	6.18
N1 (min)	40.63	5.15^#^	57.17	8.22*^$&@^	35.55	7.21^#^	34.56	4.24^#^	26.75	5.19^#^
N2 (min)	211.06	15.06	217.58	16.41	233.25	22.84	214.17	19.10	238.91	10.99
SWS (min)	71.44	9.74^#^	94.17	5.43*^@^	75.15	12.86	89.00	5.47^@^	60.40	4.36^#&^
REM (min)	91.06	3.62^@^	87.75	7.42^&@^	99.55	8.42	109.87	6.97^#^	113.12	5.61*^#^
N of awakenings	6.00	0.93^#^	12.17	1.69*^$&@^	7.00	2.24^#^	5.33	1.27^#^	5.22	1.14^#^

B-1, the day before dry immersion, DI3, DI10, DI19, on the 3rd, 10th, and 19th days of dry immersion, respectively, R + 1, 1 day after the end of dry immersion. The data are mean values and standard errors of mean. WASO, wakefulness after sleep onset; SWS, slow-wave sleep; REM, rapid eye movement sleep; N1, stage 1 of non-rapid eye movement sleep; N2, stage 2 of non-rapid eye movement sleep;%, data are presented as a percentage of time in bed. Intersession differences were examined using the post hoc Fisher LSD test, except for differences in Sleep efficiency, which were examined using Wilcoxon test. *Differences are significant relative to baseline; ^#^differences are significant relative to DI3; ^$^differences are significant relative to DI10; ^&^differences are significant relative to DI19; ^@^differences are significant relative to R + 1.

**FIGURE 2:**
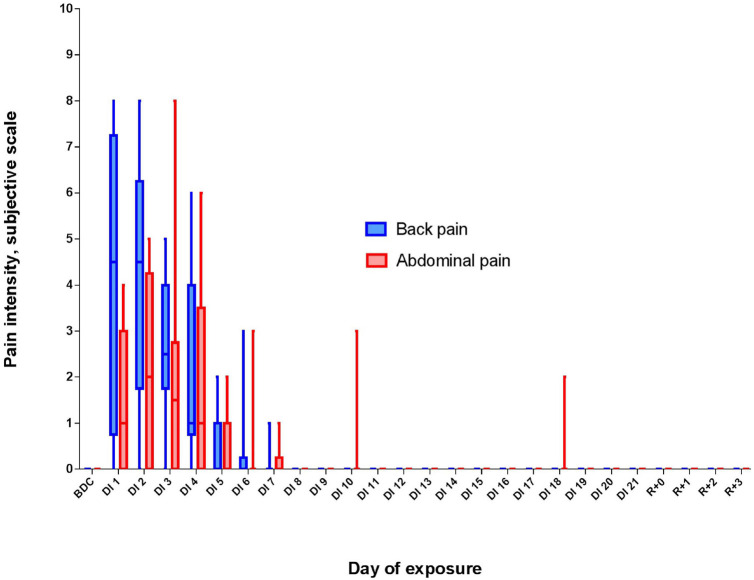
Changes in the subjective pain intensity (in points) and pain localization during 21-day Dry Immersion (DI). BDC, averaged values obtained during 7 days before DI starts; DI1, averaged values obtained on the 1st day of DI; DI2, averaged values obtained on the 2nd day of DI, etc.; R + 0, values obtained 2 h after the end of DI; R + 1, values obtained 24 h after the end of DI, etc.

To assess the impact of pain and discomfort occurred during acclimation to DI, we analyzed volunteers’ self-reports on the severity of the pain syndrome (Figure 2). The reports showed that during the first 3 days of DI, the pain intensity was the highest, reaching (for some volunteers) 8 points on a 10-point scale. However, already on the 8th day of DI, it decreased to zero for all participants and did not rise again until the end of DI.

**FIGURE 3:**
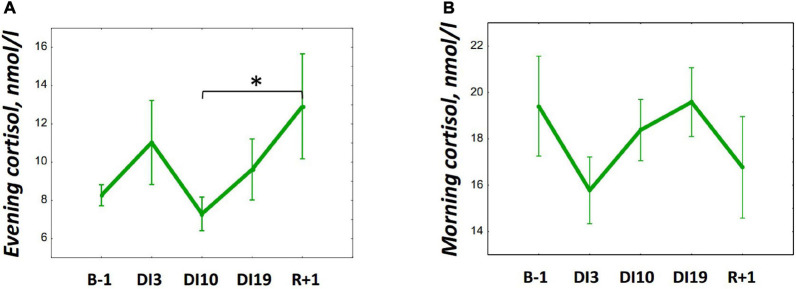
Changes in the morning **(A)** and evening **(B)** cortisol during 21-day Dry Immersion and recovery. B-1, the day before dry immersion; DI3, DI10, and DI19, on the 3rd, 10th, and 19th days of dry immersion, respectively; R + 1, 1 day after the end of dry immersion. Intersession differences were examined using the *post hoc* Fisher LSD test. ^∗^, represent significance *p* < 0.05.

The DI did not significantly affect salivary cortisol in both morning and evening samples (Figure 3). However, on R + 1, an increase in evening cortisol was found [*F*_(__4, 36)_ = 1.46, *p* = 0.234; *post hoc* compared to DI10: *p* = 0,038; also *post hoc* shows marginally significant difference relative to B-1: *p* = 0.083].

**FIGURE 4:**
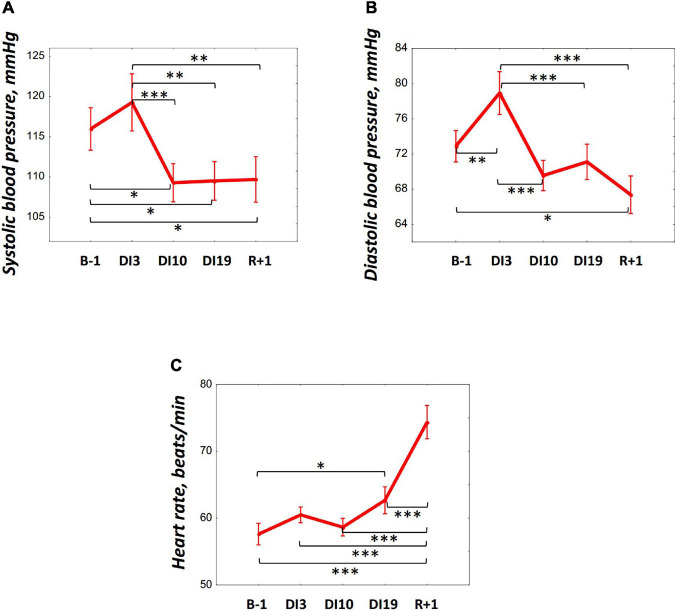
Changes in the systolic blood pressure **(A)**, diastolic blood pressure **(B)**, and heart rate **(C)** during 21-day Dry Immersion and recovery. B-1, the day before dry immersion; DI3, DI10, and DI19, on the 3rd, 10th, and 19th days of dry immersion, respectively; R + 1, 1 day after the end of dry immersion. Intersession differences were examined using the *post hoc* Fisher LSD test. ^∗^, ^∗∗^, ^∗∗∗^ represent significance *p* < 0.05, *p* < 0.01, *p* < 0.001, respectively.

The average night systolic blood pressure (SBP) significantly decreased by DI10 [*F*_(__4, 36)_ = 5.56, *p* = 0.001, *post hoc* relative to B-1: *p* = 0.021; relative to DI3: *p* = 0.001] and remained low on DI19 (relative to B-1: *p* = 0.025; relative to DI3: *p* = 0.001), and also on R + 1 (relative to B-1: *p* = 0.029; relative to DI3: *p* = 0.001) (Figure 4A). The average night diastolic BP (DBP) increased by DI3 [*F*_(__4, 36)_ = 8.73, *p* < 0.001, *post hoc* relative to B-1: *p* = 0.007] but decreased on DI10 (relative to DI3: *p* < 0.001) and continued to decrease on R + 1 (relative to B-1: *p* = 0.013; relative to DI3: *p* < 0.001) (Figure 4B). The HR averaged over the whole night did not change significantly at the beginning of DI: its values on DI3 and DI10 were comparable to the baseline values. However, there was a significant increase in HR on DI19 [*F*_(__4, 36)_ = 15.34, *p* < 0.001, *post hoc* relative to B-1: *p* = 0.045], and the most pronounced rise in HR was found on R + 1 (relative to B-1: *p* < 0.001; relative to DI3: *p* < 0.001; relative to DI10: *p* < 0.001, and relative to DI19: *p* < 0.001) (Figure 4C).

**FIGURE 5:**
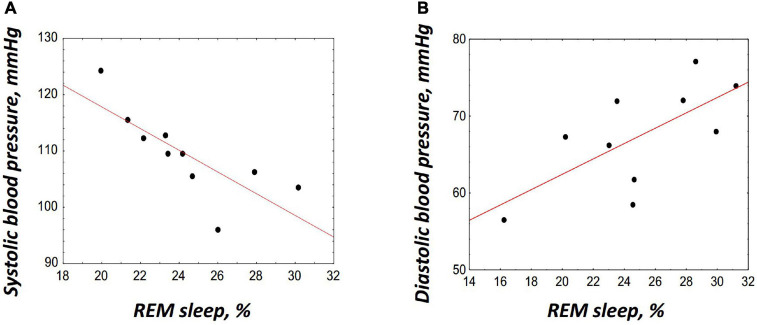
Correlations between the rapid eye movement (REM) sleep and blood pressure. **(A)** Correlation with the systolic blood pressure on DI19, *r* = –0.78, *p* = 0.008. **(B)** Correlation with the diastolic blood pressure on R + 1, *r* = 0.68, *p* = 0.031. The correlation coefficients were obtained using Pearson’s correlation analysis.

Thus, on DI19 and R + 1, i.e., on the days when an increase in the REM percentage was found, significant changes in BP, HR, and cortisol (only on R + 1) were revealed. To identify more clearly the relations between cortisol, BP, HR, and REM, Pearson’s correlation analysis was performed. The HR and cortisol did not show any significant correlations with PSG data. The BP showed opposite associations with the REM percentage on DI19 and on R + 1: a significant negative correlation was found between the SBP and REM on DI19 (*r* = –0.78, *p* = 0.008; Figure 5A); and a positive correlation was observed between the DBP and REM on R + 1 (*r* = 0.68, *p* = 0.031; Figure 5B).

## Discussion

This is the first study on the impact of DI upon sleep quality and architecture. The results suggest that during 21 days of DI and the recovery period after its end the night sleep architecture undergoes significant changes, and these changes are associated with the acclimation of the organism to the changing environment.

The most severe sleep disturbances occurred on DI3: percentages of N2 and REM decreased, whereas the sleep latency, number of awakenings, and WASO increased. This reaction, similar to the insomnia symptoms, might be elicited by the pain syndrome, particularly, by back pain. Subjective reports of the participants showed that they experienced the most severe pain at the first 3 days of DI. At this point, our data is in line with previous observations on morphological changes in the spine occurring at the first 3–4 days of DI (Kozlovskaya et al., 2015; Treffel et al., 2020). Some participants also experienced abdominal pain due to stool retention (Poliakov et al., 2020; Tomilovskaya et al., 2020). All this can provoke a lengthening of sleep latency and unstable, restless sleep with frequent awakenings.

By the 10th day of DI, when subjective pain ratings decreased to zero, most sleep architecture characteristics returned to baseline values and did not differ significantly from the B-1. However, by the end of the DI, against the backdrop of generally undisturbed sleep architecture, there was a significant increase in REM, the percentage of which increased even more at the recovery period. Compared with the non-specific sleep disturbances observed on DI3 and caused by discomfort, REM rise seems to be a specific effect of DI on sleep architecture. In this regard, our data are in line with previous observations pointing to the association of adapting to space flight and recovery from it with changes in REM. In particular, a decrease in the REM latency during the flight (Gundel et al., 1997) and after the flight (Frost et al., 1976) was observed, as well as a longer REM duration in recovery after the mission (Frost et al., 1975, 1976).

How can we explain this REM response to gravity changes? Probably, it can be a part of regulatory processes aimed at acclimating to the changing environment. In particular, changes in the cardiovascular system leading to the modification of the peripheral and central blood flows in DI and after it. As we know, during the first days of being in microgravity, there is an increase in the blood filling of the upper half of the body with an increased venous return to the right atrium (Noskov, 2011). Receptors located in this zone initiate a decrease in the circulating plasma and extracellular fluid volumes leading to body hypohydration (Leach et al., 1996; Noskov et al., 2011). Therefore, the observed increase in DBP on DI3 and its decrease on DI10 reflects the adjustment of the circulatory system to new conditions. After DI completion, the return of normal gravity and verticalization of the posture triggers the processes of readaptation aimed at increasing the volume of circulating plasma and, as a consequence, increasing BP. Thus, we can assume that REM is associated with circulatory adjustments to changed conditions.

This assumption is supported by the coincidence of the periods of REM increase with the periods of changes in BP and HR. Moreover, during these periods, namely, on DI19 and R + 1, significant correlations were found between the REM percentage and BP. Intriguingly, these associations were of the opposite sign: on the DI19 REM was negatively associated with SBP, and on the R + 1 it was positively correlated with DBP.

Our results are consistent with the data on the importance of REM for the adjustment of the body to the changing environment, in particular, there is evidence for an increase in the REM duration under stress (Suchecki et al., 2012), including immobilization stress (Rampin et al., 1991). Moreover, it was found that the REM duration was associated with successful coping with stress (Mellman et al., 2002, 2007). In addition, REM has been shown to play a significant role in brain recovery after stroke (Pace et al., 2017).

Thus, our and literature data brings us to the question of whether REM somehow facilitates the ability to adapt to varying environmental conditions. Genetic research showed that many synaptic (Ribeiro et al., 1999; Dumoulin Bridi et al., 2015) and myelin (de Vivo and Bellesi, 2019) plasticity-related genes are upregulated during REM, indicating that REM is the phase of sleep that favors adaptive modifications. On the other hand, the associations between REM and coping with stress or adapting to changed conditions can be explained in terms of the distinctive physiology of REM. This sleep phase is accompanied by intense brain activation and associated increased energy demands (Hobson et al., 2000), making REM dependent on changes in circulating glucose concentrations (Benedict et al., 2009). Thus, REM rise might be just a marker of increased energy metabolism. To answer the question of whether the cardiovascular adjustments are REM-sleep dependent or REM is just an epiphenomenon of some metabolic processes underlying these adjustments, further investigations are needed, including manipulations with REM duration (e.g., selective REM suppression) and assessing its influence on cardiovascular modifications.

If adaptive changes in the cardiovascular system are associated with an increase in the REM duration, why it was not observed on DI3? Probably, this is due to the general sleep disruption during this period caused by pain. It can be assumed that, if the pain was relieved and normal conditions for sleep initiation and maintenance would be restored, an increase in REM might be registered, including the first days of DI.

Unexpectedly, the SWS percentage, the deepest stage of sleep, which did not change throughout the DI, decreased significantly on R + 1 during the period when the most evident increase in REM was found. Deep sleep is vital for the clearance of metabolic waste from the brain (Varga et al., 2016; Ju et al., 2017), and the need for it has been shown to depend on the intensity of motor (Huber et al., 2006) and cognitive (Pugin et al., 2015) activity. Possibly, since both the DI conditions and recovery from it are accompanied by hypokinesia and a rather monotonous cognitive load, they did not increase the need for deep sleep or, under conditions of coping with physical stress, the REM phase of sleep was more important than the SWS, and the SWS/REM ratio could shift toward the latter. A decrease in SWS could also be caused by the rise in cortisol found on R + 1. It was repeatedly shown that glucocorticoids suppressed SWS (Ehlers et al., 1986; Marinesco et al., 1999), whereas REM is more resistant to glucocorticoid action (Suchecki et al., 2012). Interestingly, we did not find any significant changes in morning or evening cortisol on DI3 when our participants experienced the most severe pain. Probably, since they were informed about the discomfort associated with adapting to DI, the pain syndrome experienced was not stressful for the participants.

Some limitations of the present study should be mentioned. The use of the BP and HR values averaged over the whole night determine the first limitation of our study. Considering different sympathovagal balance during REM and NREM sleep (Ako et al., 2003), further investigations are needed for a detailed analysis of the relations between sleep and regulatory processes in the cardiovascular system. In particular, BP and HR analyses in each sleep cycle and during different sleep stages seem appropriate. The second limitation of our study is the relatively small number of subjects tested, which limits the statistical power of conducted analysis and obtained results. Third, there was an extended sleep opportunity during DI. We increased TIB to 9 h to give our participants a chance to catch up on sleep missed due to discomfort associated with the DI procedure. However, in the first days of DI, this extra time was not enough, and on DI3 in some participants, we found short sleep episodes after lights on. Unfortunately, during the DI exposure, it is difficult to standardize TIB. Such specifics of the DI procedure as being in a reclining position and limited motor activity all provoke sudden involuntary falling asleep, especially when the person is sleep-deprived. Finally, although our results suggest that cardiovascular adaptations are associated with an increase in REM duration, future studies are needed to clarify the relationship between these processes.

## Conclusion

The results suggest that the most severe sleep disturbances occur on DI3 and might be elicited by pain, particularly, back pain. On DI10, when subjective pain ratings decreased to zero, the sleep architecture returned to the baseline values. However, on the DI19, the REM duration increased and continued to rise on R + 1. Compared with non-specific changes observed on DI3, this growth was found against a background of undisturbed sleep and was associated with changes in the cardiovascular parameters. Thus, our data suggest that an increase in REM can be a part of regulatory processes aimed at adapting to the changing environment. Considering the opposite correlations between REM and the BP parameters on the DI19 and R + 1, we may assume that cardiovascular adjustments are associated with REM regardless of their direction.

## Data Availability Statement

The raw data supporting the conclusions of this article will be made available by the authors, without undue reservation.

## Ethics Statement

The study was performed according to the Declaration of Helsinki on research involving human participants. The Ethics Committee of the Institute of Biomedical Problems (Moscow) approved the study protocol (protocol no. 483 dated August 3, 2018). Written informed consent was obtained before the study procedures began, and participants were allowed to withdraw at any time. Written informed consent was obtained from the individual(s) for the publication of any potentially identifiable images or data included in this article.

## Author Contributions

EB, YU, GK, and ET conceived and designed the study. EB, YU, GK, YY, and SP were involved in the acquisition of the data. EB, YU, GK, GV, IR, and AO analyzed polysomnographic, cortisol, pain self-reports, and cardiovascular data and performed statistical analysis. EB, YU, GK, GV, NO, and ET drafted the manuscript. EB, YU, GK, GV, ET, and OO revised the manuscript critically. All authors have given final approval of the version to be submitted.

## Conflict of Interest

The authors declare that the research was conducted in the absence of any commercial or financial relationships that could be construed as a potential conflict of interest.

## Publisher’s Note

All claims expressed in this article are solely those of the authors and do not necessarily represent those of their affiliated organizations, or those of the publisher, the editors and the reviewers. Any product that may be evaluated in this article, or claim that may be made by its manufacturer, is not guaranteed or endorsed by the publisher.
