# Supplementary material for: Sleep in 21-Day Dry Immersion. Are Cardiovascular Adjustments Rapid Eye Movement Sleep-Dependent?
Source: Front Physiol. 2021 Oct 26;12:749773. doi: 10.3389/fphys.2021.749773 (PMC8576394; doi:10.3389/fphys.2021.749773)
Supplement: Supplementary file 1 [file Data_Sheet_1.docx]

**Supplementary**

**A schedule of a typical day preceding polysomnography**

| 08.00 – | Lights on |
| --- | --- |
| 08.05 – | Saliva collection |
| 08.15 – | Medical check-up |
| 09.00 – | Blood collection |
| 09.15 – | Completing a psychological questionnaire |
| 09.30 – | Hygienic procedures |
| 09.45 – | Breakfast |
| 10.30 – | The task on visual-manual tracking and visual-vestibular function |
| 11.15 – | The study of Auditory evoked potentials |
| 12.15– | Completing a psychological questionnaire |
| 14.00 – | Lunch |
| 15.00 – | The study of voluntary movement accuracy using hand dynamometry |
| 15.30 – | The task on visual perception |
| 16.30 – | Medical check-up, measuring body temperature |
| 17.00 – | Time for rest (reading, watching TV) |
| 19.15 – | Dinner |
| 21.15 – | Medical check-up |
| 21.30 – | lifting out of the bath for 20 min for hygienic procedures |
| 22.30 – | Preparing for nocturnal polysomnography |
| 23.00 – | Lights out |
